# Association of Reproductive Autonomy and Rates of State-Level Racialized Disparities in Preterm Birth and Low Birthweight

**DOI:** 10.1089/heq.2023.0060

**Published:** 2023-09-13

**Authors:** Andrew S. Bossick, Emily C. Williams, Ian Painter, Jodie G. Katon

**Affiliations:** ^1^ Department of Health Systems and Population Health, School of Public Health, University of Washington, Seattle, Washington, USA.; ^2^ U.S. Department of Veterans Affairs Health Services Research and Development Center of Innovation for Veteran-Centered and Value-Driven Care, Veterans Affairs Puget Sound Healthcare System, Seattle, Washington, USA.; ^3^ Department of Public Health Sciences, Henry Ford Health, Detroit, Michigan, USA.; ^4^ Washington State Department of Health, Olympia, Washington, USA.; ^5^ Health Services Research and Development (HSR&D) Center for the Study of Healthcare Innovation, Implementation, and Policy, VA Greater Los Angeles Healthcare System, Los Angeles, California, USA.

**Keywords:** neonatal outcomes, state laws, reproductive autonomy, disparities

## Abstract

**Introduction:**

Reproductive policies' impact on disparities in neonatal outcomes is understudied. Thus, we aimed to assess whether an index of reproductive autonomy is associated with black-white disparities in preterm birth (PTB) and low birthweight (LBW).

**Methods:**

We used publicly available state-level PTB and LBW data for all live-births among persons aged 15–44 from January 1, 2016, to December 31, 2018. The independent measure was an index of state laws characterizing each state's reproductive autonomy, ranging from 5 (most restrictive) to 43 (most enabling), used continuously and as quartiles. Linear regression was performed to evaluate the association between both the index score (continuous, primary analysis; quartiles, secondary analysis) and state-level aggregated black-white disparity rates in PTB and LBW per 100 live births.

**Results:**

Among 10,297,437 black (*n*=1,829,051 [17.8%]) and white (*n*=8,468,386 [82.2%]) births, rates of PTB and LBW were 6.46 and 8.24 per 100, respectively. Regression models found that every 1-U increase in the index was associated with a −0.06 (confidence interval [CI]: −0.10 to −0.01) and −0.05 (CI: −0.08, to −0.01) per 100 lower black-white disparity in PTB and LBW rates (*p*<0.05, *p*<0.01), respectively. The most enabling quartiles were associated with −1.21 (CI: −2.38 to −0.05) and −1.62 (CI: −2.89 to −0.35) per 100 lower rates of the black-white disparity in LBW, compared with the most restrictive quartile (both *p*<0.05).

**Conclusion:**

Greater reproductive autonomy is associated with lower rates of state-level disparities in PTB and LBW. More research is needed to better understand the importance of state laws in shaping racialized disparities, reproductive autonomy, and birth outcomes.

## Introduction

Preterm birth (PTB) and low birthweight (LBW) are leading causes of infant mortality in the United States, accounting for an estimated 25% of all neonatal deaths.^1^ Overall, PTB (gestation <37 weeks) and LBW (weight <2500 grams) affect roughly 1 in 10 and 1 in 12 births, respectively.^2^ Both PTB and LBW are also associated with short-term (e.g., infections) and long-term (e.g., developmental delay) health consequences and are standard population measures of maternal and child health.^3–5^

Although rates of PTB and LBW have decreased in recent years, striking disparities persist across racialized groups for both outcomes.^1,6,7^ For example, in 2013, the PTB rate for black infants was roughly 6 percentage points higher than in white infants (16.3% vs. 10.2%) and the rate of LBW was similarly elevated among black versus white infants (13.1% vs. 7.0%).^7^ Observed racialized disparities in PTB and LBW may partially explain widening inequality in infant mortality, with black infants having a 2.3 times higher mortality rate than white infants. These disparities in PTB and LBW and infant mortality endure even after accounting for geography, income and income inequality, poverty, educational level, and insurance status and are at least partially reflective of and sharing the same root causes as similar racialized disparities observed in maternal morbidity and mortality.^8–10^

Structural racism, a system created by Western colonization,^10^ refers to how policies, practices, laws, and societal norms result in differential access to allocation of resources based on race and reinforces and perpetuates racialized inequity and is likely the “fundamental cause” of racialized disparities in the United States.^11^ Laws at the state level are a tangible manifestation of structural racism. Therefore, investigation of the influence of state-level laws on racialized disparities in infant outcomes can help understand potential “fundamental causes” of these outcomes and related disparities, as a foundation for further work to address such upstream factors.

State laws can be used to enable or restrict reproductive autonomy, or the ability of an individual to freely decide about and control contraceptive use, pregnancy, and childbearing.^12^ Reproductive autonomy is legislated through access to contraceptives, sexual health information and education, and health services (e.g., prenatal care). Evidence from individual state policy analyses suggests that individual reproductive autonomy enabling policies are associated with overall lower rates of PTB and LBW.^13–15^ Furthermore, one study that used a composite index of state laws related to reproductive autonomy found that black women from states with more restrictive reproductive laws were more likely to give birth to LBW infants compared with black women from states with more enabling laws.^16^ In addition, some enabling policies, such as Medicaid expansion, have been shown to reduce black-white racialized disparities in PTB and LBW in states that expanded Medicaid versus those that did not.^17^

However, research that incorporates more comprehensive measures of states' reproductive autonomy environment is needed to understand state policies' impact on racialized disparities in neonatal outcomes.^18–20^

Therefore, our objective was to examine if a composite state-level index of reproductive autonomy, developed using a Delphi panel review of existing state-level laws and validated as a predictor of severe maternal morbidity and pregnancy-related mortality,^21^ was associated with state-level black-white racialized disparities in PTB and LBW. We hypothesized that greater state-level reproductive autonomy measured by our composite index of state-levels laws would be associated with decreased state-level racialized disparities in PTB and LBW.

## Methods

We used data for 45 states for PTB and all 50 states for LBW from the Centers for Disease Control and Prevention (CDC), National Center for Health Statistics natality file, for all live births to U.S. resident mothers aged 15–44 between January 1, 2016, and December 31, 2018. We accessed CDC data through CDC Wonder Online Database; data are derived from checkboxes on birth certificates.^22,23^ CDC suppresses subnational data estimates with fewer than 10 cases.^24^ Therefore, states with fewer than *N*<10 cases of PTB (*N*=5; Idaho, Montana, South Dakota, Wyoming, and Vermont) were excluded from the analysis. The University of Washington Institutional Review Board reviewed and deemed this work as exempt non-human subjects research.

### Measures

The independent variable was a composite policy index of state-level policies that characterize the level (restricting to enabling) of each state's reproductive autonomy. The composite policy index was created by first using a Delphi panel of reproductive health experts to determine state policy categories that affect reproductive autonomy. A total of eight policy categories were included: Medicaid expansion, family planning, abortion-related, comprehensive sex education, confidentiality of medical procedures, prenatal care, occupational-related, and poverty programs. Second, state-level policies for each category were collected from publicly available data, for example, women's health policy-related nonprofit organizations. One hundred six state-level laws were collected. Third, individual policies were determined to be either enabling (assigned +1 if present) or restrictive (assigned −1 if present). Lastly, the sum of the total restrictive policies for each state was then subtracted from the sum of enabling policies for each state.

Lower scores on the composite policy index represent more reproductive autonomy restrictive states, and higher scores represent more enabling states. Full development details are described elsewhere.^21^ The composite policy index was operationalized both as a continuous variable and as a categorical variable, in which the index was grouped into quartiles from the most restrictive quartile (1) to the most enabling quartile (4). The primary model used the continuous composite policy index and the secondary model assessed composite policy index as quartiles to enable analyses of a potentially nonlinear association between the index and our outcomes.

The two outcomes included in this study were state-level black-white disparities in PTB and LBW. PTB was defined as follows: (1) gestation <37 weeks measured by obstetric/clinical gestation estimate and (2) calculated based on last menstrual period. LBW was defined as neonate weight <2500 g, as indicated on birth certificate data. Maternal race was used as a proxy for infant race. Both outcomes were generated by aggregating race-specific rates (calculated by dividing the total number of outcomes in one racial group by the total number of births in that same racial group) and then subtracting the white outcome rates from the black outcome rates. State-level racialized disparity rates were calculated out of 100 live births, with lower rates indicating a smaller racialized disparity.

### Analysis

We performed simple linear regression to evaluate the association of the reproductive autonomy composite policy index and the index quartiles with racialized disparities in PTB and LBW. No adjustment variables were used due to concerns that they acted as mediators on the causal pathway or were associated with the outcome only.^25,26^ Robust standard errors were used to account for heteroscedasticity. The omnibus *F*-test was used to estimate a difference in means. We considered two-sided *p*<0.05 to be statistically significant. All analyses were performed with Stata 15.1.^27^

## Results

During the study period there were 10,297,437 black (*n*=1,829,051, 17.8%) or white (*n*=8,468,386, 82.3%) births in the 50 U.S. states (Table 1). The overall rates of PTB and LBW were 6.46 per 100 and 8.24 per 100, respectively. Compared with white births, black births had higher overall rates of PTB (obstetric/clinical gestation estimate: 14.1 vs. 4.93; last menstrual period: 14.9 vs. 5.22 per 100) and LBW (12.5 vs. 7.10 per 100).

**Table 1.** Rates of Preterm Birth and Low Birthweight per 100 Live Births, by Race and Quartile of Composite Policy Index

	Total	Black	White
	***N*** (%)	***N*** (%)	***N*** (%)
Births	10,297,437 (100%)	1,829,051 (17.8%)	8,468,386 (82.3%)

	**Mean (SD) per 100**	**Mean (SD) per 100**	**Mean (SD) per 100**
Preterm birth (*N*=45; obstetric/clinical gestation estimate)	6.46 (1.39)	14.1 (2.33)	4.93 (0.62)
Quartiles of composite policy index			
First quartile	6.98 (1.56)	14.4 (1.80)	5.11 (0.55)
Second quartile	6.99 (1.29)	15.5 (1.35)	5.16 (0.46)
Third quartile	6.18 (1.30)	13.0 (2.81)	4.91 (0.77)
Fourth quartile	5.65 (1.02)	13.4 (2.47)	4.54 (0.53)
Preterm birth (*N*=45; last menstrual period)	6.86 (1.65)	14.9 (2.66)	5.22 (0.75)
Quartiles of composite policy index			
First quartile	7.50 (1.88)	15.3 (2.20)	5.45 (0.63)
Second quartile	7.49 (1.52)	16.5 (1.90)	5.54 (0.57)
Third quartile	6.54 (1.56)	13.8 (3.18)	5.16 (0.98)
Fourth quartile	5.91 (1.12)	13.9 (2.52)	4.73 (0.51)
Low birthweight	8.24 (1.21)	12.5 (2.08)	7.10 (0.82)
Quartiles of policy index			
First quartile	8.46 (1.53)	12.9 (2.23)	7.10 (0.58)
Second quartile	8.71 (1.02)	13.8 (1.93)	7.30 (0.52)
Third quartile	8.16 (1.23)	12.9 (1.88)	7.33 (1.30)
Fourth quartile	7.60 (0.66)	11.2 (1.61)	6.66 (0.44)

SD, standard deviation.

**FIG. 1.**
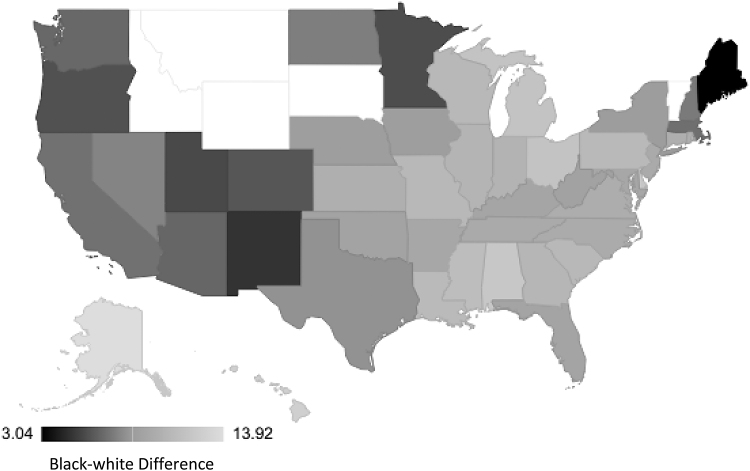
State-level racial disparity rates in preterm birth by obstetric/clinical gestation estimate. Idaho, Montana, South Dakota, Wyoming, and Vermont are missing.

**FIG. 2.**
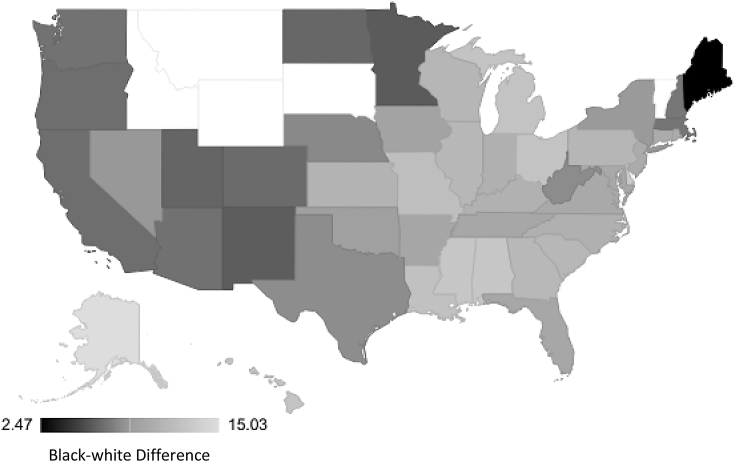
State-level racial disparity rates in preterm birth by last menstrual period. Idaho, Montana, South Dakota, Wyoming, and Vermont are missing.

**FIG. 3.**
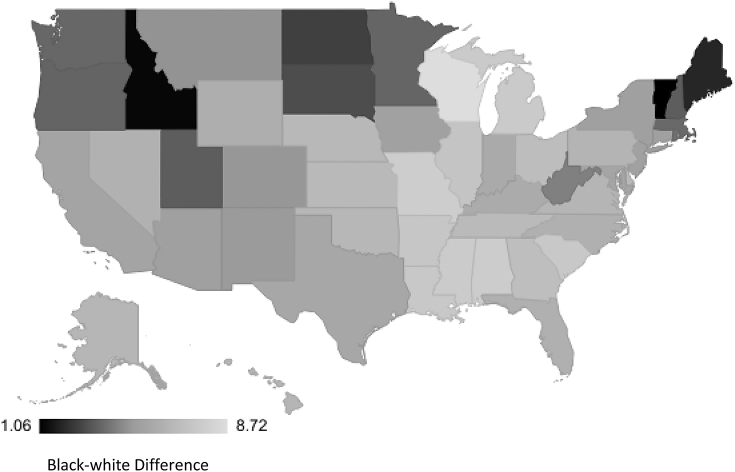
State-level racial disparity rates in low birthweight.

The rates of racialized disparities in PTB and LBW varied by state. The black-white racialized disparity in PTB by obstetric/clinical gestation estimate ranged from 3.04 (Maine) to 13.9 (Alaska) per 100 live births (Fig. 1). Similar findings were observed for PTB estimated by last menstrual period (2.47 [Maine] to 15.0 [Alaska] per 100; Fig. 2). The black-white disparity in LBW ranged from 1.06 (Vermont) to 8.72 (Wisconsin) per 100 live births (Fig. 3).

In regression models, every 1-U increase in the composite policy index was associated with −0.05 (95% CI: −0.09 to −0.01) and −0.06 (95% CI: −0.10 to −0.01) per 100 lower likelihood of black-white racialized disparity in PTB by an obstetric/clinical gestation exam and last menstrual period, respectively (Table 2). In secondary analyses when the composite policy index was entered into the model as a categorical variable based on quartiles, there was no observable association for obstetric/clinical gestation examination or last menstrual period (omnibus test=0.14, 0.24, respectively).

**Table 2.** Association of State-Level Reproductive Autonomy Composite Policy Index with Racial Disparities in Preterm Birth and Low Birthweight per 100 Live Births, 2016–2018

	Preterm birth (***N***=45) (obstetric/clinical gestation estimate)	Preterm birth (***N***=45) (last menstrual period)	Low birthweight (***N***=50)
	Coefficient (95% CI)	Coefficient (95% CI)	Coefficient (95% CI)
Composite policy index, continuous	−0.05 (−0.09 to −0.01)^*^	−0.06 (−0.10 to −0.01)^*^	−0.05 (−0.08 to −0.01)^**^
Composite policy index, quartiles	*F*>0.14	*F*>0.24	*F*>0.05
First quartile	Referent	Referent	Referent
Second quartile	0.38 (−1.02 to 1.78)	0.12 (−1.56 to 1.81)	−0.14 (−1.65 to 1.37)
Third quartile	−1.49 (−3.57 to 0.60)	−1.61 (−3.89 to 0.67)	−1.21 (−2.38 to −0.05)^*^
Fourth quartile	−0.82 (−2.57 to 0.93)	−1.10 (−2.86 to 0.66)	−1.62 (−2.89 to −0.35)^*^

Analyses are unadjusted.

^*^ *p*<0.05; ^**^p<0.01.

CI, confidence interval; *F*, omnibus *F*-test.

In models assessing LBW, every 1-U increase in the composite policy index was associated with −0.05 (95% CI: −0.08 to −0.01) per 100 lower rates of the black-white racial disparity in LBW. In addition, the most enabling quartiles (3 and 4) were associated with −1.21 (95% CI: −2.38 to −0.05) and −1.62 (95% CI: −2.89 to −0.35) per 100 lower rates of the black-white racialized disparity in LBW compared with the most restrictive quartile (1), whereas the second most restrictive quartile (2) was no different than the most restrictive quartile (1) (95% CI: −1.65 to 1.37).

## Discussion

In this national cross-sectional study, we used a previously developed composite policy index of state-level reproductive autonomy^21^ to assess its associations with state-level black-white racialized disparities in PTB and LBW. Consistent with our hypothesis, states with the most enabling reproductive autonomy environment were also more likely to have smaller racialized disparities in PTB and LBW compared with the most restrictive states. However, even in the most enabling states, disparities in adverse infant outcomes across racialized groups remained. Findings suggest that state laws that enable reproductive autonomy may be one lever via which to increase equity in birth outcomes.

Racialized disparities in health in the United States are shaped by prior and existing structural racism, including public health policy and medical care.^28,29^ Many reproductive-related policies that are restrictive were specifically designed to limit or disproportionately impact the reproductive autonomy of women of color. For example, the Hyde Amendment with few exceptions bans federal funds for abortion services, including Medicaid, a joint federal- and state-funded program, as well as coverage through insurance plans for federal employees, service members, and Veterans, all groups that disproportionately include people of color.^30,31^ Furthermore, using the Hyde Amendment as a roadmap, 32 states and Washington, DC, additionally prohibit the use of state funds toward abortion services.^31^ Similarly, state-level policies such as punitive laws regarding substance use during pregnancy frequently disproportionately target women of color.^32^

Our findings lend further support to the assertion that many state laws that restrict reproductive autonomy either implicitly or explicitly target the health and seek to assert control over black pregnant and birthing people and are upstream determinants of observed racialized disparities in health outcomes.^33^

Unlike prior studies that focus on individual laws (e.g., Medicaid expansion) or political ideologies and racialized disparities in birth outcomes, we focused on a comprehensive measure of reproductive autonomy that was community-informed and may better reflect the “mutually reinforcing systems” of structural racism. Prior work by Riley et al. used 135 state laws over 16 domains (e.g., abortion, criminal justice, education, environment, health and welfare, housing and transportation) to characterize state-level political orientation and found that infants born in states with left-leaning policies (i.e., liberal policy orientations) had lower odds of LBW and PTB compared with those with right-leaning policies, with the strongest associations among white births.^18^ Notably, racism is a ubiquitous structural determinant that through policy and other systems drives social determinants of health.

For example, left-leaning programs, such as Medicaid, that are intended to improve overall birth outcomes may potentially fail to consider social context and are more favorable toward white births. For instance, states with the greatest number of black residents are less likely to expand Medicaid. An additional example is laws that require Medicaid work requirements that exempt counties with high unemployment, where white people are more likely to live, but not cities with high unemployment, where black people are more likely to live.^34,35^ Notably, Medicaid expansion included a provision for prepregnancy eligibility, making mothers who were more likely to have had access to health care services before expansion (i.e., white birthing people) more likely to be covered after expansion.

In addition, in studies before the Affordable Care Act, among mothers on Medicaid, even after adjustment for maternal age, state, length of hospital stay, and C-section status, black mothers still had higher risks of preeclampsia, placental abruption, PTB, small-for-gestational-age infant, fetal death/stillbirth, and maternal death.^36^ However, post-Affordable Care Act Medicaid expansion appears to be associated with narrowing racialized disparities in maternal and infant outcomes possibly due to the emphasis on continuous coverage that includes standard preventive care (e.g., contraception and sexually transmitted infection testing).^17,37^ Yet even when fundamental causes of disease (e.g., health care) are addressed, new mechanisms may arise that continue to keep health socially patterned and disparities present.^38,39^

For example, although the present-day Indian Health Service has reduced health disparities for American Indian and Alaskan Native peoples through increased access, vaccination, and other services, and the Indian Healthcare Improvement Act (guarantees health care to American Indians and Alaskan Natives) is permanently authorized through the Affordable Care Act, the HIS is continually underfunded and under resourced through legislation.^40^ Furthermore, the groups with the most privilege and advantages may be able to better capitalize on new innovations and policies.^38,39^

Although our study has many strengths, for example, using a comprehensive measure of reproductive autonomy informed by a diverse group of community stakeholders, results should also be interpreted in light of its limitations. First, our independent variable comprised a diverse set of policies covering multiple reproductive policy categories; however, it is possible we have not included all the policy-level determinants that impact reproductive autonomy particularly for other minoritized populations, such as coverage of assistive reproductive technology for LGBTQIA+ birthing people. Furthermore, our composite policy index does not allow for differentiating length of exposure to a policy, where fully implemented policies may have a different effect than new policies. Second, this study was cross-sectional, and we are unable to make causal conclusions; however, cross-sectional studies serve as a strong foundation for more in-depth subsequent studies.

Third, we are not able to account for maternal state-to-state mobility, which may lead to misclassification of the reproductive autonomy environment of some individuals in this study. Finally, our measures of PTB were limited in sample size, which may impact our ability to detect a difference using our composite policy index as quartiles.

## Implications for Policy

Our research highlights that the restrictive or enabling nature of the composite effect that state-level laws have on racialized disparities in PTB and LBW are important modifiable factors to consider when evaluating neonatal birthing outcomes and future policy development. Importantly, adverse neonatal outcomes often begin preconception and during pregnancy, and thus, policy initiatives that improve the reproductive autonomy of birthing people should be prioritized. Given the already alarming rates of PTB and LBW and racialized disparities in these key population health metrics, and the leaked Supreme Court of the United States draft opinion,^41^ it is expected that these rates will increase with a greater number of high-risk pregnancies being brought to term.

It is critical that policymakers and clinicians be informed of the importance of such laws on care seeking behavior, access, and use of reproductive health services and their impact on maternal and child health. Furthermore, policymakers, funders, and researchers must identify and remove barriers to the conduct of policy research that highlights barriers to and outcomes of restricted access to critical health services.

## Conclusions

State-level racialized disparities in LBW and PTB are impacted by reproductive autonomy determined by state-level policies. Our findings suggest that greater reproductive autonomy is associated with lower rates of state-level racialized disparities. These findings suggest that the effect of structural racism through policy, a fundamental cause of health disparities, may partially drive observed disparities and provide preliminary foundation for policy reform in more restrictive states. More research is needed that investigates additional neonatal outcomes and incorporates advanced methods and study designs to better understand the mechanisms underlying the relationship between state reproductive autonomy environment and birth outcomes, including longitudinal analyses that account for policy year and state-level effects. Importantly, future research and policy work should be centered on equity by prioritizing the impact on reproductive autonomy and outcomes of programs and policies, for example, Temporary Assistant for Needy Families or Medicaid coverage through 12 months postpartum, that primarily serve low-income people of color. Black women and their families rely on these programs because of historical and present-day racism that has resulted in income and education inequality. These and similar programs act as a safety net to prevent further disadvantages and adverse health outcomes.

## Abbreviations Used

CDC: Centers for Disease Control and Prevention
CI: confidence interval
LBW: low birthweight
PTB: preterm birth
